# Evaluation of platelet-rich plasma injection activity in the treatment of abnormal uterine bleeding

**DOI:** 10.4274/tjod.93709

**Published:** 2019-01-09

**Authors:** Gökçe Turan, Pınar Yalçın Bahat, Alev Aydın, Bahar Yüksel Özgör

**Affiliations:** 1 University of Health Sciences, Kanuni Sultan Süleyman Training and Research Hospital, Clinic of Obstetrics and Gynecology, İstanbul, Turkey; 2 İstanbul Esenler Obstetrics and Children Diseases Hospital, Clinic of Obstetrics and Gynecology, İstanbul, Turkey

**Keywords:** Abnormal uterine bleeding, growth factors, platelet-rich plasma

## Abstract

**Objective:**

To evaluate the effectiveness of intracavitary platelet-rich plasma (PRP) therapy in patients diagnosed as having abnormal uterine bleeding (AUB).

**Materials and Methods:**

A total of 149 patients with AUB were included in the study. Seventy-four of these patients were included in the study group and 75 were included in the control group. All patients were evaluated using transvaginal ultrasonography. Endometrial curettage was performed to exclude underlying organic pathologies. The study group underwent intracavitary PRP therapy. Both patient groups were called for follow-up at the end of the third month. Their endometrial thickness and amount of bleeding (pictogram and pads/day) were evaluated using transvaginal ultrasonography.

**Results:**

There was no statistically significant difference between the study and control groups in terms of the decrease in the amount of bleeding. In addition, there was no statistically significant difference between the two groups in terms of the increase in endometrial thickness.

**Conclusion:**

In this study, it was observed that intracavitary PRP therapy did not make a significant difference in the decrease in the amount of bleeding and in the increase in endometrial thickness between the study and control groups.

**PRECIS:** This study was conducted to investigate the effect of platelet rich plasma on abnormal uterine bleeding and endometrial thickness.

## Introduction

Abnormal uterine bleeding (AUB) is the bleeding of organic or non-organic causes that indicate irregularity in the amount, duration, and frequency of menstrual bleeds^(1,2)^. AUB constitutes approximately one-third of the reasons for referral to hospital in gynecology practice. The acronym polyp; adenomyosis; leiomyoma; malignancy and hyperplasia; coagulopathy; ovulatory dysfunction; endometrial; iatrogenic; and not yet classified, which classifies uterine bleeding abnormalities by bleeding pattern and etiology in reproductive-age women, was published by the International Federation of Gynecology and Obstetrics in 2011. The term menorrhagia was later replaced by heavy menstrual bleeding, and the term metrorrhagia was replaced by intermenstrual bleeding^(3,4,5)^. It can be treated medically or surgically. The idea of using platelet-rich plasma (PRP) for therapeutic purposes has emerged because platelets contain high amounts of growth factors and are the main source of growth factor complexes that play a key role in the wound healing process. PRP is defined as plasma fraction of autologous blood having a platelet concentration above baseline^(6,7)^. PRP is a cellular component of plasma, which is obtained by centrifuging whole blood, and contains a higher platelet concentration than whole blood. The cellular component of plasma is normally composed of 93% red blood cells, 6% platelets, and 1% leukocytes. PRP contains about 3-5 times the number of platelets found in normal blood circulation. PRP is basically a reversal of ratio between red blood cells and platelets; and is made up of 93% platelets, 6% red blood cells, and 1% leukocytes^(8)^. Enzyme-linked immunosorbent assay and immunoprecipitation studies have shown that there is a 7-fold increase in transforming growth factor-b, a 30-fold increase in platelet-derived growth factor, and a 10-fold increase in epidermal growth factor^(9)^. *In vitro *and animal studies have demonstrated that PRP positively affects gene expression, matrix synthesis, and vascularization in tendon cells^(10)^. Today, it is used mostly in surgical procedures such as orthopaedic interventions, dental and oral surgery, traumatic surgical procedures, maxillofacial surgery, spinal surgery, heart bypass surgery, angiogenic interventions, sliding flap operations, and surgical repair of macular lesions and corneal epithelial defects. Although it is not used very frequently in the field of gynaecology, it is used particularly in infertility treatment and in patients with recurrent miscarriages, as well as in postoperative wound treatment. It has been observed that PRP therapy for infertility issues increases endometrial growth and improves pregnancy outcomes in patients with a thin endometrial lining^(11)^. In summary, it has been concluded that PRP has a regulatory role on endometrial inflammation and thereby provides a rapid proliferation process and maintains endometrial tissue integrity in the long term^(12)^. However, there are still few studies in the field of gynecology in the literature. In this study, we aimed to investigate the effect of intracavitary PRP injections on the amount of bleeding and endometrial thickness at the end of 3 months in patients with AUB aged between 20 and 40 years.

## Materials and Methods

Our study was performed prospectively after approval was obtained from the Local Ethics Committee of University of Health Sciences Kanuni Sultan Süleyman Training and Research Hospital. A total of 149 patients, who were admitted to the obstetrics and gynecology outpatient clinic due to AUB between March 1^st^, 2017, and June 1^st^, 2017, were included in the study. Reproductive-aged patients aged between 20 and 40 years and who had AUB resistant to drugs were included in the study. Patients who did not fit the age range, who had hormonal disturbances and additional systemic diseases, and who had endometrial pathology (polyp, myoma) were excluded from the study. One hundred sixty patients were randomly divided into two equal groups of 80 each: the study group and the control group. Six of the 80 patients in the study group and 5 of the 80 patients in the control group were excluded from the study because they did not come to follow-up in the third month. During the evaluation phase, the uterine cavity was first evaluated using transvaginal ultrasonography. The amount of bleeding (pictogram and pads/day) was recorded. The double-wall endometrial thickness was measured in the sagittal plane. Then, hormone markers [follicle-stimulating hormone (FSH), leutinizing hormone (LH), estradiol (E_2_), and thyroid-stimulating hormone (TSH)] were analyzed. After clinical evaluation, 74 patients who were randomized by the computer program underwent a complete curettage. A total of 30 cc venous blood was collected into 3 EDTA tubes from these patients using a butterfly needle. The blood samples were centrifuged at 3200 rpm for approximately 15 minutes. The PRP fraction (buffy coat), which was separated from the whole blood by centrifugation and remained on the tube, was pulled and collected using a syringe needle. Approximately 3-4 cc PRP was obtained from 30 cc venous blood. The prepared PRP material was introducced to the uterine cavity using a cannula within 10 minutes from collection. The patients were kept in bed for about 10 minutes. A total of 75 patients in the control group underwent a complete curettage after clinical evaluation and no additional intervention was performed. The patients were discharged after their general condition was stabilized and called to return for follow-up at the end of the third month. Both groups were asked to keep a diary of bleeding for 3 months. They were called for follow-up in the follicular phase of the menstrual cycle 3 months later. Bleeding issues observed in the patients after 3 months were questioned and recorded. The double-wall endometrial thickness was measured using transvaginal ultrasonography.

### Statistical Analysis

All results are expressed as mean +/- standard deviation. The relationships between the variables were assessed using Pearson’s correlation test. The one-sided Kolmogorov-Smirnov test was used to assess the distribution of the data. Student’s t-test was used to compare normally distributed data between the two groups. The chi-square test was used to examine categorical data. Two-way analysis of variance was used to assess the effects on the independent variables in the study and control groups. A p value of <0.05 was considered statistically significant. All statistical analyses were performed using the SPSS statistical software, version 18.0 (SPSS Inc., Chicago, IL, USA).

## Results

The mean age of the study group was 26.87±5.40 years, and the mean age of the control group was 28.19±4.93 years (p=0.420). The demographic characteristics of the two groups are shown in Table 1. As seen in Table 1, when the demographic data of the study and control groups were compared, the two groups were similar in terms of age, body mass index, gravidity, parity, abortion, FSH value, and amount of bleeding at first admission (pads/day). Endometrial thickness was evaluated in the follicular phase of the menstrual cycle using transvaginal ultrasonography at the end of the third month in the control and study groups. The mean endometrial thickness was 7.8 mm in the study group and 9 mm in the control group. No statistically significant difference was found between the two groups (Table 2). The amount of bleeding at the end of the third month was evaluated based on the number of pads/day in the control and study groups. The mean amount of bleeding at first admission was 7 pads/day in the study group and 6 pads/day in the control group (Table 1). As seen in Table 3, it was found that the two groups were similar in terms of the amount of bleeding at the end of the third month. There was no statistically significant difference between the two groups in terms of the amount of bleeding at the end of the third month.

## Discussion

AUB accounts for 10-15% of gynecologic problems. It is most commonly seen at the beginning (after menarche) and the end (perimenopausal period) of the reproductive life cycle (70%). Fifty percent is seen after the ages of 40 years and 20% is seen during adolescence. The remaining 30% is seen in the reproductive period. One out of every 20 women aged between 30-49 years is admitted to hospital due to AUB. There is no pathologic reason for approximately half of these. PRP is used especially for postoperative wound healing in gynecology. Moreover, it is injected into the uterine cavity in infertile patients or in patients with recurrent miscarriages and thus it benefits from growth factors in its content^(11,13,14)^. The age range of our study was selected as 20-40 years. The effect of PRP on AUB was examined particularly in reproductive-aged patients. Patients who had previously received various medical treatments for AUB but continued to report problems despite these medical treatments were included in the study. FSH, LH, E_2_, and TSH values were analyzed in the patients. Patients with polycystic ovary syndrome, low ovarian reserve, and thyroid dysfunction were not included in the study. Furthermore, patients with additional systemic diseases and who had organic uterine pathologies were excluded from the study. The two groups were similar in terms of demographic data and the amount of bleeding at first admission. The main reason why the patients in the study group underwent a complete curettage prior to PRP therapy was to increase the endometrial blood flow except for pathologic diagnosis and to allow the diffusion of PRP into the endometrium. Although there are not many publications related to PRP in the field of gynecology in the literature, it has been tested in assisted reproductive technique (ART) treatments. In our study, intracavitary PRP injection was applied as used in ART treatments. Patients were evaluated in terms of decreases in the amount of bleeding and increases in endometrial thickness. Significantly different results were obtained in PRP studies using ART. In the pilot study of Zadeh-Modarres et al.,^(15)^ 10 patients with history of inadequate endometrial growth in frozen-thawed embryo transfer cycles were evaluated. Intrauterine infusion of PRP was performed in these patients. They reported that all patients had increased endometrial thickness and some patients had chemical and clinical pregnancies. On the contrary, our study found that although endometrial thickness at the end of the third month was thinner in the study group than in the control group, there was no statistically significant difference between the two groups. In the study of Tandulwadkar et al.,^(14)^ PRP was administered to the uterine cavity in 68 patients who had recurrent cycle cancellation due to endometrial insufficiency and received ART treatment. PRP was used to achieve the optimal endometrial thickness in these patients. It was concluded that endometrial thickness increased from 5 mm to 7.2 mm in patients who underwent PRP therapy in the cycle before the procedure. In the same study, patients with a thin endometrium who were treated with PRP were evaluated before and after treatment using Doppler ultrasonography, which revealed increased tissue vascularization. Based on these findings, they reported that PRP enhanced angiogenesis and had a proliferative effect on the endometrium^(14)^. Chang et al.^(11)^ showed a similar effect in their study. PRP injection was administered to patients who were scheduled for *in vitro *fertilization (IVF) treatment on the 10^th^ day of hormone replacement therapy prior to the embryo transfer. The cut-off value of endometrial thickness on the 7^th^ day (embryo transfer day) was considered as 7 mm, and endometrial thickness was measured using transvaginal ultrasonography. Women with endometrial thickness >7 mm underwent IVF-embryo transfer. A live pregnancy was achieved in all women who underwent PRP therapy. In contrast, our study found that the increase in endometrial thickness was not significant. In the *in vivo* study of Marini et al.^(12)^ performed in the bovine endometrium, it was concluded that PRP had a regulatory role on endometrial inflammation and thereby provided a rapid proliferation process and maintained endometrial tissue integrity in the long-term. In the study of Challen et al.,^(16)^ it was mentioned that stem cell studies on the endometrium clearly revealed the effect of stem cells on the regenerative process and that growth factors supported this process. The regenerative process progressed rapidly and the tissue integrity quickly returned to normal because the PRP we used was similarly rich in growth factors. However, this process ultimately did not reflect on the decrease in the amount of bleeding and the increase in endometrial thickness. In the study of Hang-Yong Jang et al.^(17)^, endometrial injury was first achieved in 60 female rats using ethanol. PRP therapy was applied after 72 hours, and tissue sampling was performed in the mid-luteal phase. Subsequently, there was an increased rate of proliferation of endometrial tissue stained with hematoxylin and eosin and Masson’s trichrome staining. Pathologic data have proven that PRP therapy enhances regeneration. In our study, the ethanol-induced endometrial damage was mechanically achieved through complete curettage, and then PRP injection was performed. However, the proliferation level did not reach statistical significance in the study group compared with the control group. On the contrary, endometrial thickness was found to be lower in the study group than in the control group.

Our study has some limitations. The patients received only a single dose of PRP. Measurements were only made in 3 months after administration. Therefore, the effect of repeated doses of PRP and its longer-term effect could not be assessed. Longer-term studies are needed to investigate whether PRP provides tissue integrity and regeneration in the long term.

## Conclusion

As a result of this study, it was determined that there was no statistically significant difference in the amount of bleeding between the group that had AUB and underwent PRP therapy and the control group. Unlike previous studies, it was seen that the increase in endometrial thickness was less in the PRP-treated group than in the control group. There are many unanswered questions about the composition of PRP, the characteristics of individual blood products, different production protocols, different application methods, and effect mechanisms applied at the cellular level by PRP and its individual components. Although there are few publications in the literature about the effects of PRP on the endometrium, this relationship can be explained more clearly by future studies.

## Figures and Tables

**Table 1:**
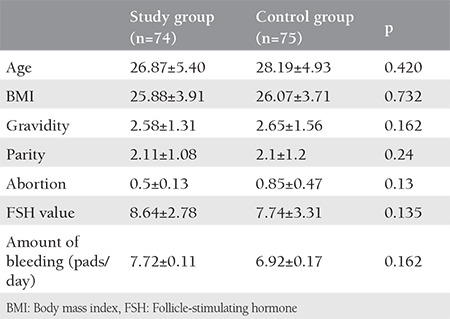
Demographic characteristics of the study and control groups

**Table 2:**
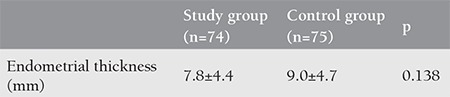
Endometrial thickness values at the end of the third month in the study and control groups

**Table 3:**
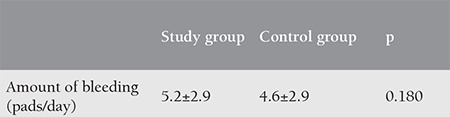
The amount of bleeding at the end of the third month in the study and control groups
